# A Case Report of a Sclerotic Fibroma of the Oral Mucosa

**DOI:** 10.7759/cureus.27627

**Published:** 2022-08-03

**Authors:** Luísa Figueiredo, Paula Maria Leite, Margarida Varela, Filipa Veiga, Ana Fernandes

**Affiliations:** 1 Oral Surgery, Centro Hospitalar Universitário de Lisboa Central, Lisbon, PRT; 2 Pathology, Centro Hospitalar Universitário de Lisboa Central, Lisbon, PRT; 3 Pediatric Stomatology, Hospital Dona Estefânia - Centro Hospitalar Universitário de Lisboa Central, Lisbon, PRT

**Keywords:** benign fibrous tumor, oral mucosa, storiform collagenoma, sclerotic fibroma, cowden syndrome

## Abstract

Sclerotic fibroma, also known as storiform collagenoma, is a rare benign tumor that grows on the skin, but it can also appear, less frequently, in the oral mucosa. It can present as part of Cowden syndrome manifestation, especially when multiple lesions are encountered, but it may also appear as a solitary, sporadic lesion in healthy individuals. We describe a patient, diagnosed with Cowden syndrome, who presented with a sclerotic fibroma in the oral mucosa, which is a very uncommon manifestation of Cowden syndrome.

## Introduction

Sclerotic fibroma (SF), also called storiform collagenoma, is a rare benign tumor [1]. It presents as a cutaneous fibrous neoplasm [2], and it is especially unusual in the head and neck mucosal tissue [1-2]. It derives from the proliferation of fibroblasts that exhibit increased production of type-I collagen [1]. SF affects both sexes, with a slight female predominance [2].

It presents as either solitary or multiple skin nodules, being an important marker in patients with Cowden syndrome (CS) [3-5], or as a sporadic, small, solitary cutaneous mass in healthy individuals [5].

First described in 1963 by Lloyd and Dennis [4], Cowden syndrome, or multiple hamartoma syndrome [1], is a rare autosomal-dominant inheritance genodermatosis [6], characterized by multiple hamartomas of ectodermal, mesodermal, and endodermal origins [4-6]. The phosphatase and tensin homolog (PTEN) gene negatively regulates cell proliferation and cell cycle progression [1].

CS is characterized by a large range of systemic abnormalities [1,3]: patients usually have macrocephaly, trichilemmomas, and papillomatous papules/hamartomas [1]. CS is associated with a large range of PTEN hamartoma tumor syndrome (PHTS), which is a group of disorders characterized by the disorganized growth of native cells in native tissues [1]. CS is the only PHTS disorder associated with a well-reported predisposition to malignancies [1,4]; they have a higher probability of developing benign and malignant tumors of the mucous membranes and genitourinary and gastrointestinal tracts [1,3].

Mucocutaneous lesions are present in 99 to 100% of cases [5] and are pathognomonic [4]. The characteristic mucocutaneous findings include a variety of benign and malignant neoplasms (including SF) of the skin (face, acral, and palmoplantar) [3]. Fibroepithelial hyperplasia or diffuse papillomatosis are oral lesions frequently found in patients with Cowden syndrome [3,7], and often involve sites such as the tongue, gingiva, and lips [3,8]. On the contrary, SF is rarely identified at the same site [1,3,7].

The estimated prevalence of Cowden Syndrome is 1/200,000 [4]. CS is most often diagnosed during the third decade of life (the late 20s) [1,4].

There are very few reported cases of SF within the oral mucosa [1,3,5,6,9]. To our knowledge, only nine SFs of the oral cavity have been published in the medical literature.

The finding of multiple SFs should motivate further investigations due to the probability of a Cowden syndrome diagnosis (chromosomal analysis for mutation in the suspected PTEN gene) [1], and once the diagnosis is confirmed, further clinical and familial history investigations are recommended due to the reported increased risk of malignancies.

## Case presentation

A 42-year-old man presented in the Stomatology department, with a known diagnosis of Cowden syndrome. A genetic study revealed an intronic mutation - thymine (T) to guanine (G) - T>G - transition at the +32 position of intron 8.

**Figure 1:**
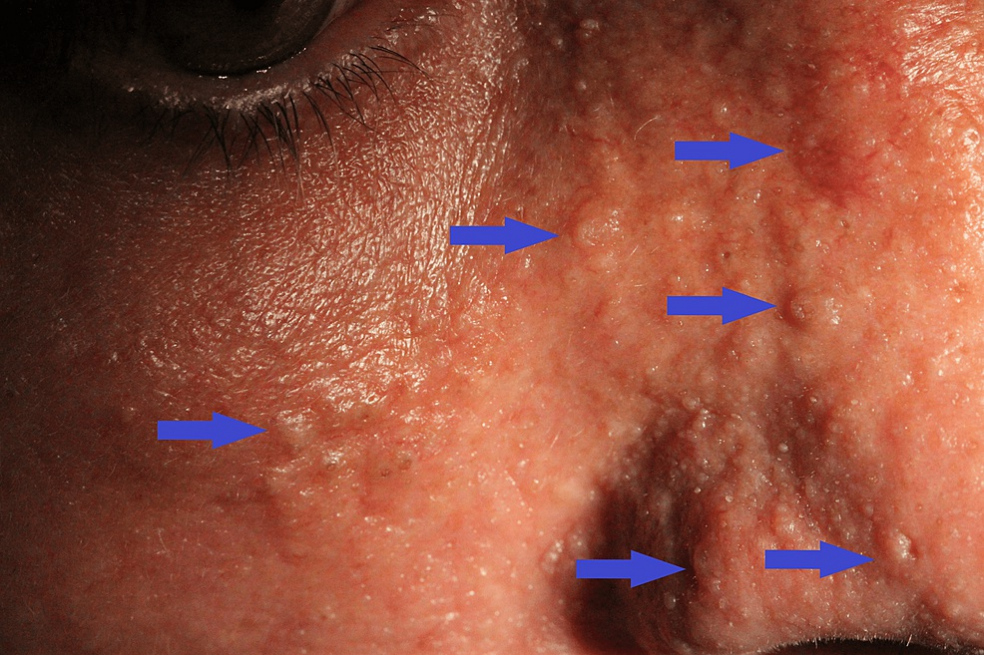
Patient's face and nose, presenting multiple hamartomas and trichilemmomas (blue arrows)

He has been followed by our Dermatology department since 2012, due to multiple hamartomas and trichilemmomas, mostly on the face and scalp (Figure 1).

Despite many appointments, the patient never showed up to perform the required colonoscopy, thyroid ultrasound, and genetic study.

He presented in the Stomatology department with an 11-month history of a slow-growing, painless mass on the dorsal surface of the tongue. The patient was referred with no history of trauma.

**Figure 2:**
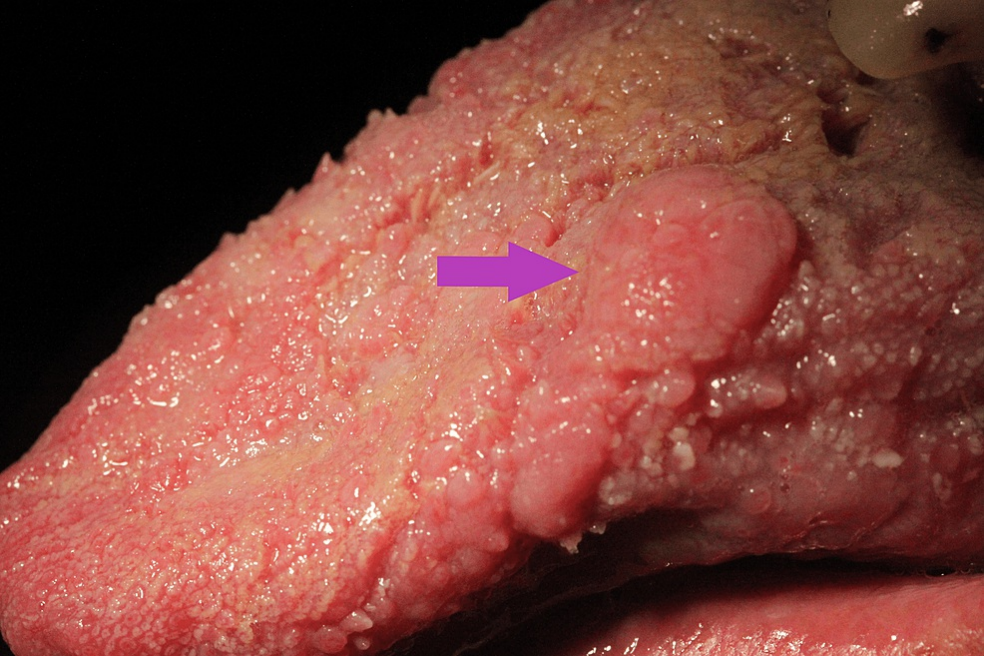
Lesion A - Pedunculated firm mass on the dorsum of the tongue (purple arrow)

**Figure 3:**
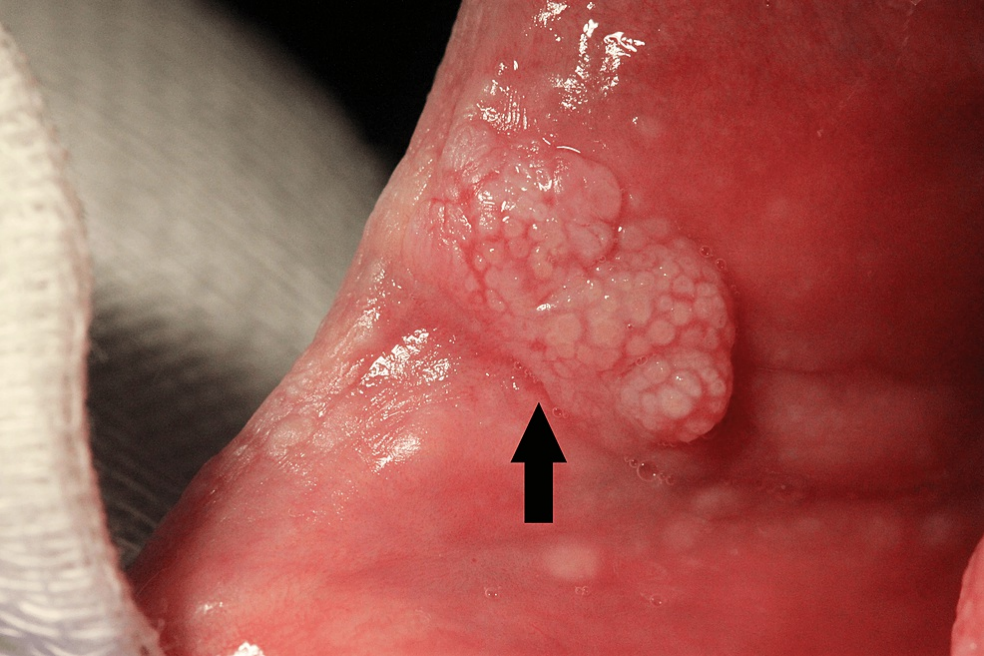
Lesion B - White papillomatous lesion at the left commissure of the lips (black arrow)

**Figure 4:**
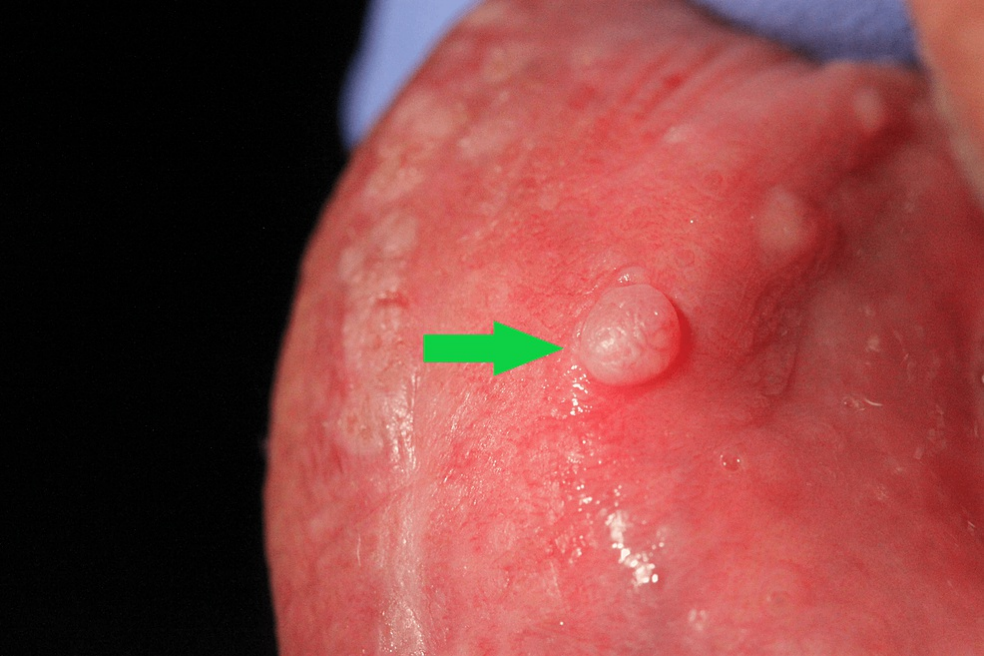
Lesion C - Sessile lesion on the lower lip (green arrow)

Clinical examination showed a pedunculated firm mass that measured about 2 cm on the dorsal surface of the tongue - lesion A (Figure 2). We also noted a white papillomatous lesion at the left commissure of the lips - lesion B (Figure 3) - and a sessile lesion on the wet mucosa of the lower lip - lesion C (Figure 4).

A panoramic radiograph excluded abnormalities, and a fibroepithelial hyperplasia clinical diagnosis was assumed.

Surgical excision was performed due to the small size of the lesions. Macroscopically, they presented as well-circumscribed, firm, and mucosa-colored.

**Figure 5:**
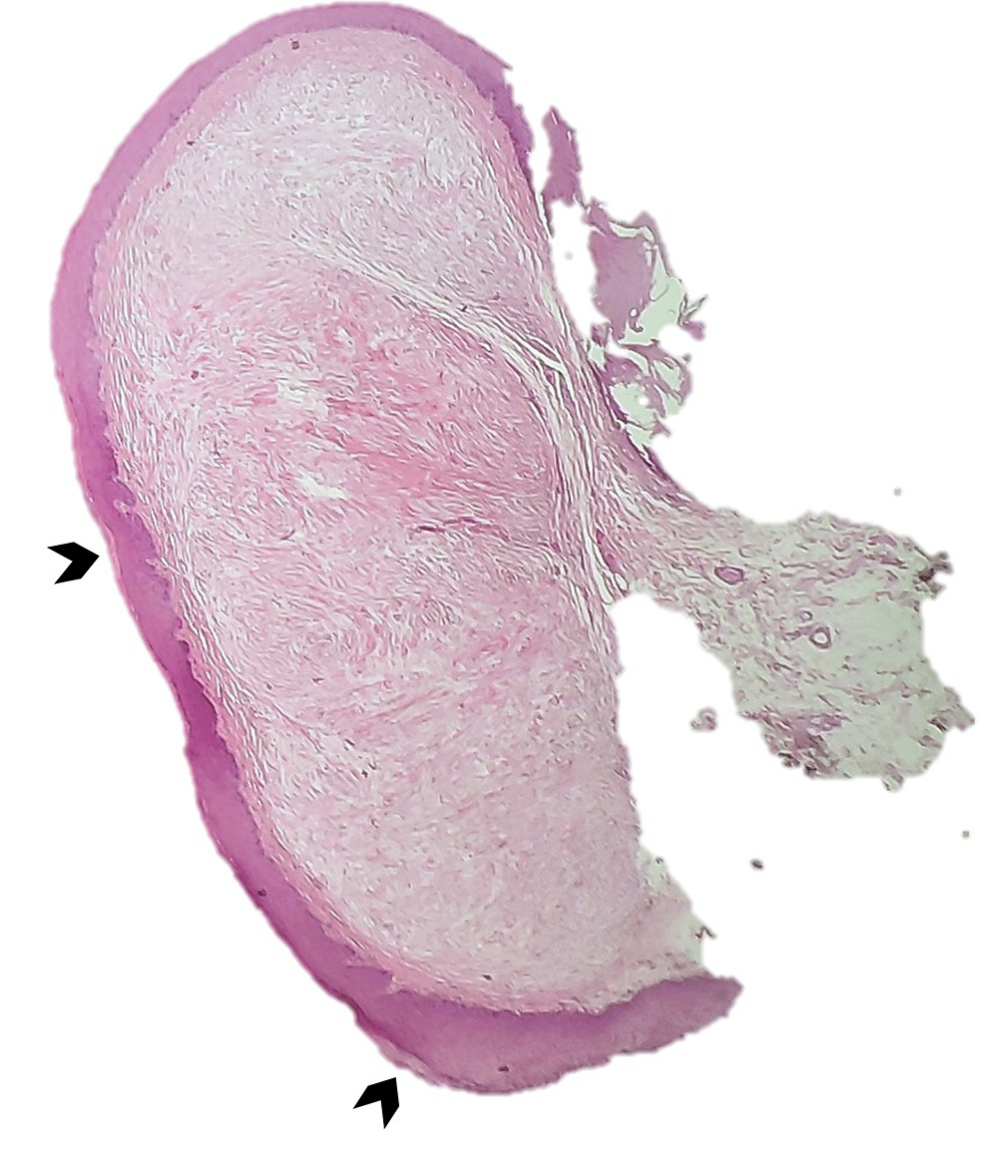
A non-capsulated hypocellular nodule located in the lamina propria, covered by squamous epithelium with areas of keratinization (dark bold arrow) Hematoxylin & eosin at 20x magnification

**Figure 6:**
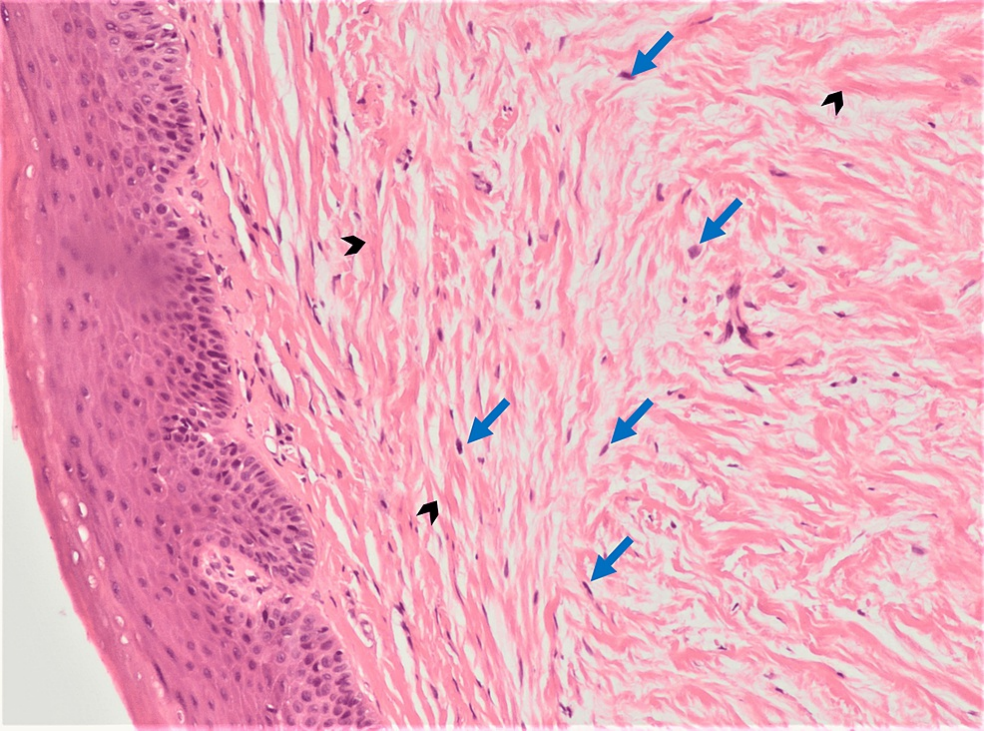
Eosinophilic collagen fibers (dark bold arrow) separated by prominent clefts, along with scattered fibroblasts (blue arrow) Hematoxylin & eosin at 100x magnification

**Figure 7:**
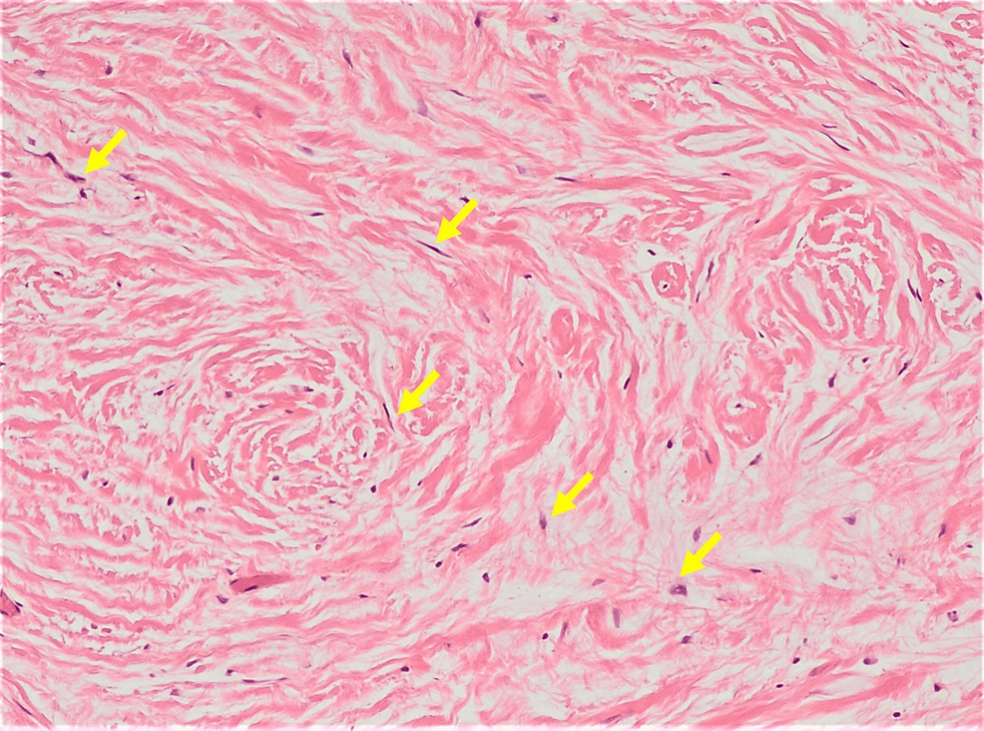
Scattered fibroblasts (yellow arrow) are bland and mono-nuclear, presenting a spindle or stellate morphology Hematoxylin & eosin at 100x magnification

Histopathologic examination showed that lesion A was characterized by a non-capsulated proliferation of fibrous tissue located in the *lamina propria* (Figure 5) consistent with sclerotic fibroma: a well-limited, hypocellular nodule consisting of eosinophilic collagen bundles, arranged in a storiform or concentrically lamellar pattern and separated by a prominent spectum, along with scant fibroblasts (Figure 6) with variable spindle or stellate morphology (Figure 7); lesion B was compatible with a hamartomatous lesion and lesion C with reactive fibroepithelial hyperplasia.

## Discussion

A sclerotic fibroma is a rare benign tumor of the soft tissue, which usually presents as a well-circumscribed mass in the skin [1-2]. There are less than 100 case reports of SF in the world literature [10]. It is very uncommon for SF to manifest on the oral mucosa [3], but this kind of presentation has been published before [1]. SF may manifest as either a sporadic, small, solitary cutaneous mass in otherwise healthy individuals [5] or as a solitary or multiple skin nodules, being an important marker in patients with Cowden syndrome (CS) [3-5].

Cowden syndrome is a genetic disorder characterized by a broad range of symptoms and signs, including multiple cutaneous/mucocutaneous lesions and involvement of different organs [4]. The organ system that most consistently manifests this syndrome is the skin [5]. Contrary to our male patient, there is a slight female predominance of CS [2].

According to the consulted literature, and as reported in this case, oral lesions, such as fibroepithelial hyperplasia and hamartomatous or diffuse papillomatosis (lesions B and C) are frequently found in patients with Cowden syndrome. We found it worth reporting the finding of an oral Sclerotic fibroma (lesion A).

Albeit the histomorphology is usually representative, the neoplastic SF cells usually stain for vimentin and stain positively for CD34 in about 60% of cases [1,3]. In this case, the tumor cells strongly expressed vimentin but they were negative for CD34.

There are a few fibrous lesions that should be included in the histologic differential diagnosis of tumors in the oral cavity [1,4] such as solitary fibrous tumor, traumatic fibroma, giant cell fibroma, and benign fibrous histiocytoma [8]. The fibroblasts of traumatic fibroma, giant cell fibroma, and benign fibrous histiocytoma do not react with anti-CD34 antibodies [8].

Cowden syndrome is associated with a higher risk for benign and malignant tumors of the thyroid, breast, kidney, and endometrium [1]. As such, the involvement of multiple sites of SF in a patient should motivate further investigations due to the great probability of Cowden syndrome diagnosis [1] (chromosomal analysis for mutation in the suspected PTEN gene, on chromosome 10q23 [2]). Once the diagnosis is confirmed, as it was in this case, further clinical and familial history investigations are recommended [1]. In this patient, such investigations were not yet performed, due to lack of compliance.

These tumors can be treated with surgical resection; occasionally, they may show recurrence - a recurrence time from 2.5 years to 7 years after removal has been reported [10].

## Conclusions

Circumscribed sclerotic fibroma is a rare benign tumor that is uncommonly found in the oral mucosa. This case report shows a very rarely identified oral SF that was found in a patient with a previous diagnosis of Cowden syndrome. The diagnosis of SF was established by histopathology, after excision of the lesions (with free margins). Even though it can appear as a solitary, isolated skin lesion, syndromic associations, such as Cowden syndrome, should be excluded, especially when multiple sites of SF are involved.

The oral cavity can be the first noticeable manifestation of systemic disease and should not be neglected when performing a physical examination of the patient.

## References

[1] Elledge R, Nandra B, Bates T, Zardo D, Parmar S (2020). Storiform collagenoma (sclerotic fibroma) of the oral mucosa. Br J Oral Maxillofac Surg.

[2] Stocchero GF (2015). Storiform collagenoma: case report. Einstein (Sao Paulo).

[3] Alawi F, Freedman PD (2004). Sporadic sclerotic fibroma of the oral soft tissues. Am J Dermatopathol.

[4] Porto AC, Roider E, Ruzicka T (2013). Cowden syndrome: report of a case and brief review of literature. An Bras Dermatol.

[5] Guimarães P, Branco A, Carvalho E (2002). Síndrome de cowden: relato de um caso [Article in Portuguese]. An Bras Dermatol.

[6] Yehia L, Eng C (2001). PTEN hamartoma tumor syndrome. GeneReviews® [Internet].

[7] González-Vela M, Carmen MD, Val-Bernal Val-Bernal (2004). Solitary sclerotic fibroma of the oral mucosa. Am J Dermatopathol.

[8] Lee JH, An JS, Lee ES, Kwon SY, Kim YS (2007). Comparison of sporadic sclerotic fibroma and solitary fibrous tumor in the oral cavity. Yonsei Med J.

[9] Ide F, Mishima K, Saito I (2003). Solitary sclerotic fibroma of the lip. Br J Dermatol.

[10] Lira-Valero FJ, Carrillo-Cisneros ER, Pulido-Díaz N, Quintal-Ramírez MJ, Godínez-Aldrete L (2020). Circumscribed storiform collagenoma, an unusual tumor. Dermatol Online J.

